# Impact of urban agglomeration construction on urban air quality–empirical test based on PSM–DID model

**DOI:** 10.1038/s41598-023-42314-8

**Published:** 2023-09-12

**Authors:** Wanxiong Zhang, Ruiyun Cui, Changyuan Li, Hailong Ge, Zhuoya Zhang, Xueqiong Tang

**Affiliations:** 1 College of Geography and Ecotourism, Southwest Forestry University, Kunming, 650224 China; 2 Ecological Civilization Research Center of Southwest China, National Forestry and Grassland Administration, Southwest Forestry University, Kunming, 650224 China

**Keywords:** Climate sciences, Environmental social sciences

## Abstract

Urban agglomerations have become a new trend in the development of urbanization and regionalization in the world today. The construction of urban agglomerations has brought rapid economic development as well as a series of ecological and environmental problems, especially the impact on urban air quality. How to understand and evaluate the impact of urban agglomeration construction on air quality is a key issue that requires attention. City cluster construction is equivalent to a "quasi-natural experiment". This study empirically examines the impact of urban agglomeration construction on air quality in southwest China by constructing a PSM–DID model. It is found that: (1) City cluster construction has significantly improved urban air quality in urban clusters with lagging and forward-looking effects on air quality. (2) In terms of influencing factors, the level of economic development considerably improves the air quality of urban cluster cities, the industrial structure severely deteriorates the air quality of these cities, and meteorological factors highly affect their air quality. Among them, average annual urban rainfall significantly reduces urban air pollutant concentrations in urban clusters, average annual temperature significantly increases urban air pollutant concentrations, and average annual wind speed can reduce urban air pollutant concentrations. (3) Urban agglomerations are spatially heterogeneous in their impact on air quality. In this context, the topographical conditions and the level of development of urban agglomerations have a non-negligible influence on pollutant concentrations. (4) The distribution pattern of air quality pollutant concentrations in each urban agglomeration is unstable, and there are large differences in these concentrations between different urban agglomerations.

## Introduction

Urban agglomerations are an important spatial form of integrated regional development. However, their development may cause several environmental problems. The Chinese government has established a green strategy for urban agglomeration construction to achieve clean transformation by enhancing firm green innovation^1^. The Chinese government has proposed a total green transformation of economic and social development through the 14th 5-Year Plan period^2^. However, the development of urban agglomerations drives economic growth but also brings some ecological and environmental problems, which have a more prominent impact on urban air quality. Urban agglomeration development and construction is considered by the World Health Organization to be the biggest health threat to air pollution in 2019^3^.

On the one hand, the concentration of population and lifestyle changes produce the "living effect" of urbanization, which leads to deterioration of air quality and puts it under greater pressure of urban air pollution ; on the other hand, the construction of urban agglomerations leads to industrial agglomeration which also helps to reduce the cost of pollution control and produces the "production effect" of urbanization. On the other hand, the construction of urban agglomerations leads to industrial agglomeration, which also helps to reduce the cost of pollution control and produce the "production effect" of urbanization, while the joint prevention and control of air pollution in different regions also helps to improve air quality^4^. At the same time, air pollution usually has cross-regional effects, so it is important to strengthen environmental management and maintain overall control of environmental quality in a specific region^5^.

 So, does the development of urban agglomerations worsen air quality or improve it? Does the impact of urban agglomeration construction on urban air quality differ from one city to another^4^? Based on the current research, there is no unified and clear argument. If the impact of urban agglomeration construction on urban air quality can be evaluated accurately, the heterogeneity of the impact of each urban agglomeration construction on air quality and its impact factors. It can provide scientific arguments for the development strategy of each urban agglomeration and urban air pollution prevention and control.

## Literature review

Current studies focus on the impacts of air pollution, as well as the causes, mechanisms, pathways, and prevention. In this context, extensive studies have been conducted by academics around the topical issue of regional air quality, the perspectives of which mainly focused on two aspects. One is the study of the spatial and temporal variation patterns of regional air quality. The second is the study of air quality impact factors. The current research progress is shown in Table 1:

**Table 1:** The current research progress.

Research perspectives		Characterization indicators	Research characteristics
Spatial and temporal variation patterns			
Time scale	Temporal scales cover daily, monthly, seasonal, and interannual evolution patterns, etc^6,7^	The air quality characterization indicators usually include individual pollutants (SO2, NO2, O3, PM10, PM2.5, etc^8–10^.) as well as combined air quality indices (API^11^, AQI^12,13^, etc.)	The effects and mechanisms of urban expansion and urban air quality pollutant concentrations are studied to reveal the characteristics of air pollution such as spatial agglomeration effect and spatial spillover effect^14^. Qualitative characterization of the spatial variation of air quality in a given year based on AQI averages^15,16^
Spatial Scale	Spatial scales include the national scale^17,18^, provincial areas, urban clusters^19,20^, and multiple typical regions such as the Yangtze River Delta, Pearl River Delta, and other hotspots^21–30^		To study the effects and mechanisms of urbanization’s impact mechanisms. Urban agglomerations face more serious problems than urban areas, but have received less attention overall^31,32^
Impact factor			
Natural factors	Meteorological factors	Dust^33^, wind speed^34^, precipitation^35^, temperature^36^, wind direction, humidity, and air pressure^4^	Have a direct impact on air quality
	Physical Geographical Factors	Topography^37^ and vegetation index^38^	
Socio-economic factors	Current scholars have explored the impact of economic growth, industrial structure^39^, urbanization^40^, industrialization^41^, land use^42^, foreign direct investment (FDI)^43^, population density^44^, urban built-up areas, population distribution, industrial development^45^ ,transportation^46^, energy structure^47^, and oil price changes^48^ on air pollution and air quality		Human socio-economic activities are the main source of air pollutant emissions and the main cause of air pollution formation. In recent years, more and more scholars have focused their attention on the influence of socio-economic factors on urban air quality^49,50^
Research methodology			
The research methods included spatial econometric models^51,52^, geographically weighted regression (GWR) models^53^, geographic probe models^54^, principal component analysis and multiple linear regression models, etc. The study of the factors influencing air pollution in the urban development process examines its relationship with air pollution to explore its path of action on air pollution. Moreover, they used difference-in-difference models to analyze the policy effects of national high-tech zones^55^, political blue sky^56^, smart city construction^57^, low-carbon city construction^58^, natural resource asset discharge audit^59^, and the development of major events^60^			

In terms of the current research status, most of the studies on regional air quality take single pollutants as the research object or analyze the spatial and temporal characteristics of multiple pollutants in a single year, and there is a relative lack of comprehensive assessment studies based on multiple pollutants in multiple years^14,16^. In the study of influencing factors, less attention has been paid to the interaction between different influencing factors^49,50^the current study mainly focuses on the better developed and larger urban clusters. There is a relative lack of research on urban agglomerations in less developed and border areas. Less attention has been paid to public policies.

Combining the current research progress, this paper focuses on two questions: Does the development and construction of urban agglomerations worsen or improve air quality? Is there any difference in the impact of different urban cluster construction on urban air quality? The time scale of the study is from 2015/1/1 to 2020/12/31; urban agglomerations in the less developed and border areas of southwest China are used as the study area. An integrated assessment study based on multiple pollutants over multiple years. Analyze the interactions between different impact factors. By using the PSM–DID model, the net effect of urban cluster construction on air pollution is identified by overcoming some estimation biases that existed in previous studies.

## Data and methodology

### Data

#### Study area

**Figure 1:**
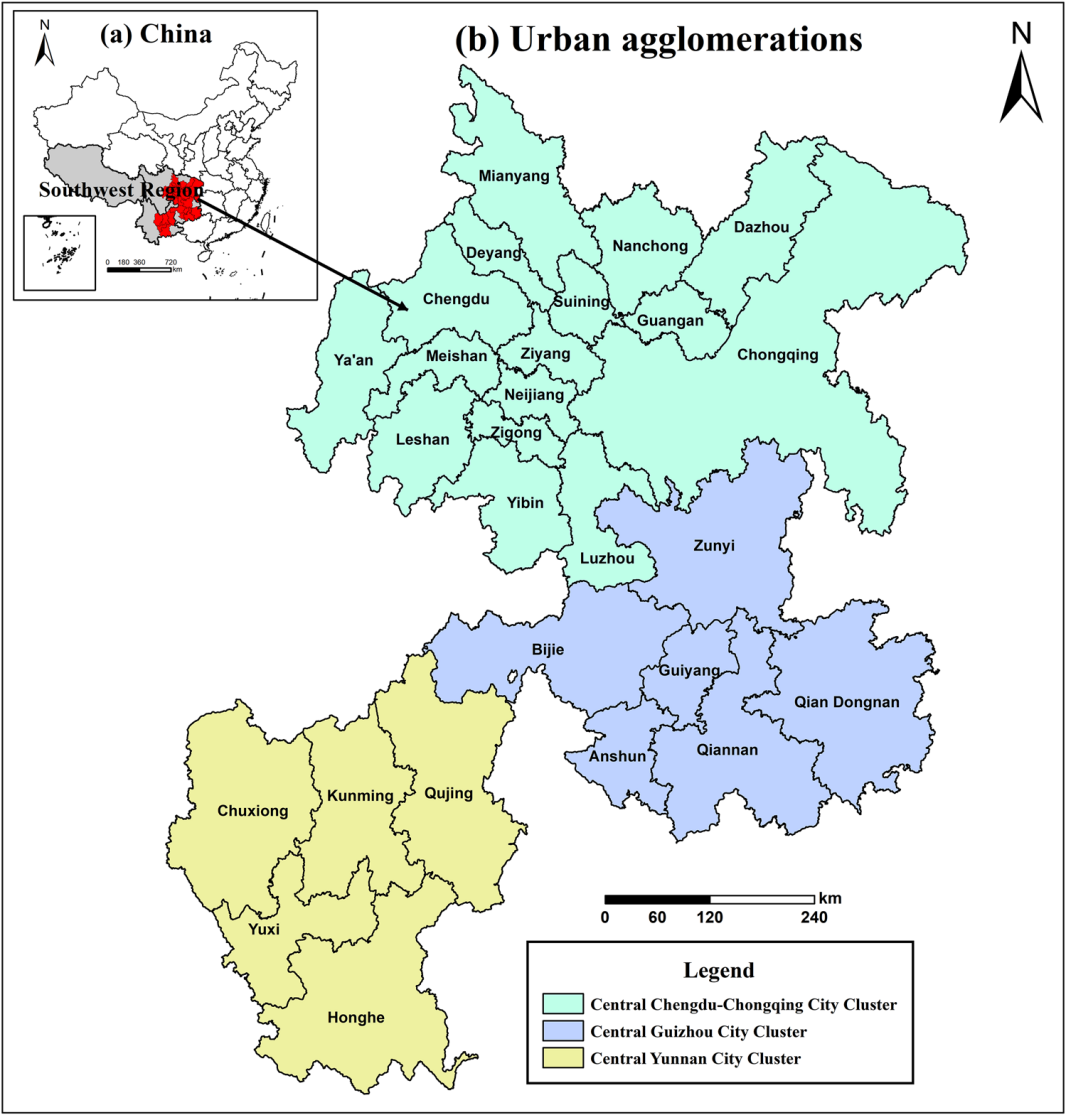
Major urban agglomerations in southwest China. The maps in this study were processed and plotted by ArcGIS 10.8. The map images we use come from Environmental Sci-ences, Chinese Academy of Sciences (http://www.resdc.cn/).

The study area of this paper is the three major urban agglomerations in southwest China. Southwest China is one of the main battlefields for China's future urbanization, with the Central Yunnan, Central Guizhou, and Central Chengdu-Chongqing urban agglomerations being key urban agglomerations fostered by the State. In this study, 27 prefecture-level cities/states radiating from the Central Yunnan City Cluster, Central Guizhou City Cluster, and Central Chengdu-Chongqing City Cluster in southwest China were used as the experimental group (see Fig. 1). The other 20 prefecture-level cities/states outside the city cluster plan served as the control group , to explore the impact of city cluster construction on urban air quality.

#### Data sources

The data time scale of this paper is from 2015/1/1 to 2020/12/31. AQI, PM10, PM2.5, CO, NO2, SO2, and O3 concentration data were obtained from the national real-time urban air quality release platform of the China General Environmental Monitoring Station (http://www.cnemc.cn/). The concentration data of the six air pollutants and the air quality index of 156 stations in 47 prefecture-level cities were finally selected through screening and collation. The time accuracy is mostly 24-h sliding average data and O3 is 8-h sliding average data. The monthly data were converted to annual data by interpolation method^4^. Meteorological factor data were obtained from provincial and municipal statistical yearbooks, statistical bulletins, and water resource bulletins, and some missing data were obtained by kriging interpolation using the daily value dataset of Chinese terrestrial climate information (V3.0) from the China Meteorological Data Network (http://data.cma.cn). The 1 km resolution meteorological data of 156 stations in 47 prefecture-level cities were selected, sorted and calculated by screening. Other socio-economic data were mainly obtained through the statistical bulletins on national economic and social development of each city and state, government work reports, statistical yearbooks, and related books. Some data were supplemented by trend extrapolation and linear interpolation.

#### Selection of variables

The explanatory variables used are the new Ambient Air Quality Standard (GB3095-2012) for the six air pollutants CO, NO2, O3, PM10, PM2.5, SO2, and AQI in Chinese cities. Among them, near-ground ozone (O3) has become one of the most harmful gases to urban air quality in China after PM2.5^61^. Air Quality Index (AQI) is a non-linear dimensionless index that quantitatively describes the air quality condition. The larger the value, the higher the level and category, and the darker the color of the representation, the more serious the air pollution condition is and the greater the health risk to human beings, and it is suitable for indicating the short-term air quality condition and change trend of a city.

As for the control variables, two categories of natural factors and socio-economic factors were selected. Natural factors focused on three meteorological factors, annual precipitation (PRE), annual mean temperature (TEM) and annual mean wind speed (WIN)^62^. These factors affect the processes of transport, dispersion, secondary pollution, deposition and dissipation of air pollutants. Precipitation and wind speed affect the wet and dry removal processes of aerosol particles^34^. The latter focused on factors related to the level of socio-economic development of the city and its industrial structure, characterized by the per capita gross regional product (PCG) and the industrial structure by the share of the secondary industry in the regional GDP (SEC) and the added value of the secondary industry (SAV). The data descriptions and descriptive statistics for the explanatory variables and a series of control variables are shown in Table 2.

**Table 2:** Variable measures and descriptive statistical analysis.

	Meaning	Unit	Average value	Standard deviation	Minimum value	Maximum value
PCG	Level of economic development	Yuan	41,117	17,570	13,097	103,757
SEC	Industrial structure	%	40.27	9.668	21	71.45
SAV		billion	807.9	1411	34.67	9992
TEM	Average annual temperature	℃	16.01	2.903	6.358	21.03
PRE	Annual precipitation	0.1 mm	11,319	1891	7653	21,182
WIN	Average annual wind speed	m/s	3.901	0.715	2.773	6.384
AQI	Air Quality Index		58.61	15.26	33.92	101
PM2.5	Fine particulate matter	μg-m^−3^	31.35	13.01	8.500	73.58
PM10	Respirable particulate matter	μg-m^−3^	51.84	18.87	14.75	107.8
SO2	Sulphur dioxide	μg-m^−3^	12.45	6.561	3.833	38.25
NO2	Nitrogen dioxide	μg-m^−3^	22.82	9.079	7.583	53.58
CO	Carbon monoxide	μg-m^−3^	0.772	0.204	0.357	1.678
O3	Ozone	μg-m^−3^	78.40	10.23	49.17	101.9

### Methodology

Estimating Policy Effects Using a Multiperiod DID Model^63^. Changes in urban air quality levels are influenced by both "time effects" and "policy effects". Currently, the difference-in-difference (DID) method is commonly used to separate the "time effect" from the "policy effect"^64^. Propensity Score Matching (PSM) is a non-parametric method of counterfactual inference^65^. By analyzing non-experimental and experimental data, it can effectively reduce selectivity bias and endogeneity, and is more commonly used in the evaluation of policy effects. Urban cluster construction is equivalent to a "quasi-natural experiment"^66^. In this study, the effect of urban cluster planning on air quality is evaluated as a policy, and the net effect of urban cluster construction on air quality is assessed using the PSM–DID model. The three major city cluster development plans in this paper were approved at different times, and the policies were implemented at different points in time^65^.In order to ensure the net effect of the urban cluster policy, the Multiperiod DID method can effectively exclude the influence of random factors on the results and mitigate the endogeneity problem^63^. However, the difference-in-difference method must satisfy the common trend assumption, otherwise it is likely to cause "selective bias" in the sample and reduce the reliability of the model results^66^. Propensity score matching (PSM) constructs counterfactual events by finding samples with similar characteristics to the treatment group from the control group, which is helpful to overcome the "selectivity bias" but difficult to overcome the endogeneity problem. The combination of PSM and DID methods can reduce the "selectivity bias" and better address the endogeneity, so that the "policy treatment effect" can be effectively identified and evaluated^65^.Subsequently, this study combined the PSM and DID for robustness estimation^67^.

#### Research model setting

In this study, urban cluster cities were defined as the experimental group and non-urban cluster cities as the control group to compare the differences in air quality between them before and after the implementation of urban cluster planning policies. The three major city cluster development plans in this paper were approved on April 12, 2016, March 8, 2017, and January 14, 2020, respectively. The policy was implemented at different points in time, and the policy grouping variables in the regional dimension and the policy phasing variables in the time dimension were generated using the multi-period double difference method (DID)^14^.Two dummy variables were constructed: (1) experimental and control dummy variables, with the experimental group being urban cluster cities, defined as 1, and the control group being non-urban cluster cities, defined as 0; (2) policy time dummy variables, defined as 1 after the approval of the urban cluster development plan and 0 before the approval. In this paper, urban cluster planning is used as a regional integration policy and its impact on economic resilience is explored using the DID approach. The baseline regression model is set as follows:^66^

1 $$Y_{it} = \partial_{0} + \partial_{1} DID_{it} + \partial_{2} X_{it} + \mu_{i} + \lambda_{t} + \varepsilon_{it}$$

2 $$Y_{it}^{PSM} = \partial_{0} + \partial_{1} DID_{it} + \partial_{2} X_{it} + \mu_{i} + \lambda_{t} + \varepsilon_{it}$$

where i denotes the city and t the year. Yit is the explanatory variable, denoting the air quality of city i in year t; DIDit is the core explanatory variable, which contains a time dummy variable and a policy dummy variable,which takes the value of 1 if city i is planning an urban agglomeration in year t and thereafter, and 0 otherwise. Xit represents the control variable, controlling variables related to air quality; μi denotes the city fixed effects; λt represents the time fixed effects; εit is the random error term and a the coefficient. The analysis and evaluation of the model are carried out using STATA software.

## Empirical analysis

### Basic regression results

To test the effect of urban cluster construction on urban air quality, Eq. (1) was estimated in this paper and the results are shown in Tables 3, 4 and 5. In the odd-column model, this study has only put the urban cluster development plan as a dummy variable as well as the explanatory variable into the model for regression, and the even-column model was based on the odd-column model with the addition of control variables affecting urban air quality.

**Table 3:** Basic regression results of the impact of the construction of the Central Yunnan Urban Agglomeration on air quality.

	(1)	(2)	(3)	(4)	(5)	(6)	(7)	(8)	(9)	(10)	(11)	(12)	(13)	(14)
	AQI	AQI	PM2.5	PM2.5	PM10	PM10	SO2	SO2	NO2	NO2	CO	CO	O3	O3
DID	− 4.186***	− 3.244*	− 2.842*	0.340	− 8.990***	− 5.037*	− 4.379***	− 1.110	− 2.257***	− 2.327**	− 0.104**	− 0.041	− 2.136	− 4.435*
	(− 2.74)	(− 1.70)	(− 1.90)	(0.19)	(− 3.80)	(− 1.75)	(− 3.66)	(− 0.87)	(− 2.98)	(− 2.51)	(− 2.63)	(− 0.90)	(− 0.93)	(− 1.68)
PCG		0.000		− 0.000**		− 0.000**		− 0.000***		0.000		− 0.000**		0.000**
		(0.16)		(− 2.28)		(− 2.18)		(− 4.20)		(0.77)		(− 2.64)		(2.45)
SEC		0.196		0.142		0.093		0.051		0.035		− 0.001		0.174
		(1.12)		(0.87)		(0.35)		(0.44)		(0.42)		(− 0.17)		(0.72)
SAV		− 0.000		− 0.002		− 0.000		− 0.002		− 0.002		− 0.000		0.002
		(− 0.15)		(− 0.63)		(− 0.09)		(− 0.99)		(− 1.17)		(− 0.56)		(0.49)
TEM		0.142		− 0.269		− 0.259		− 0.010		− 0.200		0.000		− 0.028
		(0.30)		(− 0.60)		(− 0.36)		(− 0.03)		(− 0.87)		(0.01)		(− 0.04)
PRE		− 0.000		− 0.000		− 0.000		0.000		0.000		0.000		− 0.001***
		(− 1.38)		(− 0.58)		(− 0.17)		(0.81)		(1.46)		(1.26)		(− 2.66)
WIN		− 3.365		0.257		− 3.297		− 1.989		− 1.116		− 0.083		− 4.173
		(− 1.01)		(0.08)		(− 0.65)		(− 0.89)		(− 0.69)		(− 1.03)		(− 0.90)
Constant	48.378***	57.976***	23.152***	29.578*	41.487***	66.153**	11.634***	24.585*	16.288***	20.252**	0.763***	1.216***	78.974***	94.721***
	(91.34)	(3.12)	(44.75)	(1.69)	(50.62)	(2.35)	(28.10)	(1.97)	(62.09)	(2.25)	(55.90)	(2.71)	(99.64)	(3.68)
Sample sizes	96	96	96	96	96	96	96	96	96	96	96	96	96	96
R-squared	0.087	0.130	0.044	0.158	0.154	0.232	0.145	0.403	0.101	0.180	0.081	0.233	0.011	0.196
Number of id	16	16	16	16	16	16	16	16	16	16	16	16	16	16
Controls	No	No	No	No	No	No	No	No	No	No	No	No	No	No
Year Effect	Yes	Yes	Yes	Yes	Yes	Yes	Yes	Yes	Yes	Yes	Yes	Yes	Yes	Yes

*, **, and *** represent 10%, 5%, and 1% significance levels respectively; t-values in parentheses.

**Table 4:** Basic regression results of the impact of Central Guizhou urban agglomeration construction on air quality.

	(1)	(2)	(3)	(4)	(5)	(6)	(7)	(8)	(9)	(10)	(11)	(12)	(13)	(14)
	AQI	AQI	PM2.5	PM2.5	PM10	PM10	SO2	SO2	NO2	NO2	CO	CO	O3	O3
DID	− 2.378	0.899	− 146.954**	− 111.964	− 249.642**	− 191.136	− 83.196**	− 61.792	− 105.533*	− 82.144	− 3.880*	− 2.985	− 380.412*	− 304.336
	(− 0.86)	(0.22)	(− 2.02)	(− 0.97)	(− 2.04)	(− 0.99)	(− 2.19)	(− 1.03)	(− 1.99)	(− 0.98)	(− 1.99)	(− 0.97)	(− 1.96)	(− 0.99)
PCG		− 0.000		− 0.003		− 0.005		− 0.002		− 0.002		− 0.000		− 0.007
		(− 0.40)		(− 0.39)		(− 0.39)		(− 0.49)		(− 0.38)		(− 0.39)		(− 0.34)
SEC		0.266		2.025		3.874		1.113		1.711		0.062		4.536
		(0.73)		(0.20)		(0.23)		(0.21)		(0.23)		(0.23)		(0.17)
SAV		− 0.001		− 0.108		− 0.182		− 0.053		− 0.077		− 0.003		− 0.280
		(− 0.11)		(− 0.68)		(− 0.68)		(− 0.64)		(− 0.67)		(− 0.66)		(− 0.66)
TEM		8.781		18.331		34.318		8.023		10.459		0.368		33.068
		(1.36)		(0.10)		(0.11)		(0.09)		(0.08)		(0.08)		(0.07)
PRE		− 0.001		− 0.010		− 0.017		− 0.005		− 0.008		− 0.000		− 0.025
		(− 0.69)		(− 0.32)		(− 0.33)		(− 0.30)		(− 0.34)		(− 0.31)		(− 0.31)
WIN		− 3.273		− 240.610		− 410.838		− 132.359		− 178.124		− 6.549		− 656.454
		(− 0.33)		(− 0.87)		(− 0.89)		(− 0.92)		(− 0.89)		(− 0.88)		(− 0.89)
Constant	51.202***	− 74.265	108.066***	960.705	182.750***	1,572.833	58.821***	559.530	78.190***	750.696	2.869***	27.727	282.514***	2,849.403
	(36.08)	(− 0.60)	(2.91)	(0.28)	(2.93)	(0.27)	(3.03)	(0.31)	(2.89)	(0.30)	(2.88)	(0.30)	(2.86)	(0.31)
Sample sizes	54	54	54	54	54	54	54	54	54	54	54	54	54	54
R-squared	0.016	0.169	0.085	0.133	0.087	0.136	0.098	0.149	0.083	0.130	0.082	0.129	0.081	0.125
Number of id	9	9	9	9	9	9	9	9	9	9	9	9	9	9
Controls	No	No	No	No	No	No	No	No	No	No	No	No	No	No
Year Effect	Yes	Yes	Yes	Yes	Yes	Yes	Yes	Yes	Yes	Yes	Yes	Yes	Yes	Yes

*, **, and *** represent 10%, 5%, and 1% significance levels, respectively; t-values in parentheses.

**Table 5:** Basic regression results of the influence of Central Chengdu-Chongqing Urban agglomeration construction on air quality.

	(1)	(2)	(3)	(4)	(5)	(6)	(7)	(8)	(9)	(10)	(11)	(12)	(13)	(14)
	AQI	AQI	PM2.5	PM2.5	PM10	PM10	SO2	SO2	NO2	NO2	CO	CO	O3	O3
DID	− 8.035***	2.808*	− 9.380***	0.909	− 16.406***	0.500	− 6.540***	− 1.454	0.259	2.963***	− 0.136***	0.003	2.531	3.603
	(− 3.81)	(1.69)	(− 4.77)	(0.56)	(− 5.45)	(0.24)	(− 6.13)	(− 1.49)	(0.29)	(3.23)	(− 4.10)	(0.10)	(1.26)	(1.47)
PCG		− 0.000*		− 0.000**		− 0.000**		− 0.000		− 0.000		− 0.000**		− 0.000
		(− 1.82)		(− 2.04)		(− 2.17)		(− 1.42)		(− 0.91)		(− 2.17)		(− 0.54)
SEC		0.429***		0.417***		0.897***		0.309***		0.152***		0.007***		− 0.089
		(4.53)		(4.53)		(7.45)		(5.55)		(2.90)		(4.01)		(− 0.64)
SAV		− 0.001		− 0.003		− 0.005*		− 0.001		− 0.002*		− 0.000		0.004
		(− 0.37)		(− 1.52)		(− 1.94)		(− 0.48)		(− 1.72)		(− 0.96)		(1.12)
TEM		10.238***		6.990***		9.541***		1.688		0.508		0.024		7.538*
		(3.83)		(2.69)		(2.81)		(1.07)		(0.34)		(0.48)		(1.91)
PRE		− 0.001		− 0.000		− 0.000		0.000		− 0.001***		− 0.000		− 0.000
		(− 1.11)		(− 0.40)		(− 0.59)		(0.29)		(− 3.06)		(− 1.50)		(− 0.04)
WIN		− 5.853*		− 7.271**		− 7.986**		− 3.353*		− 1.111		− 0.064		6.325
		(− 1.86)		(− 2.37)		(− 1.99)		(− 1.81)		(− 0.64)		(− 1.08)		(1.36)
Constant	75.186***	− 71.451	45.761***	− 45.059	74.001***	− 71.142	17.234***	− 9.825	28.869***	30.370	0.901***	0.748	79.280***	− 56.232
	(46.87)	(− 1.42)	(30.62)	(− 0.92)	(32.32)	(− 1.11)	(21.24)	(− 0.33)	(41.96)	(1.09)	(35.89)	(0.79)	(51.95)	(− 0.76)
Sample sizes	132	132	132	132	132	132	132	132	132	132	132	132	132	132
R-squared	0.118	0.659	0.173	0.650	0.214	0.759	0.256	0.611	0.001	0.358	0.134	0.518	0.014	0.079
Number of id	22	22	22	22	22	22	22	22	22	22	22	22	22	22
Controls	No	No	No	No	No	No	No	No	No	No	No	No	No	No
Year Effect	Yes	Yes	Yes	Yes	Yes	Yes	Yes	Yes	Yes	Yes	Yes	Yes	Yes	Yes

*, **, and *** represent 10%, 5%, and 1% significance levels respectively; t-values in parentheses.

The regression results of the Central Yunnan City Cluster, Central show that the coefficients of the dummy variables of urban agglomeration construction, AOI, PM10, SO2 and NO2 are significantly negative at the 1% level, CO is significantly negative at the 5% level, and PM2.5 is significantly negative at the 10% level, indicating that urban agglomeration construction significantly reduces urban PM10, SO2, NO2, CO and PM2.5 concentrations and AQI; while O3 regression results are not significant, indicating that the level of socioeconomic development has no significant effect on O3.

From the regression results of the control variables, the socio-economic factors, the socio-economic development level has a significant impact on the urban air pollutant concentration and the coefficient is negative, indicating that the socio-economic development level significantly reduces the urban air pollutant concentration and the AQI index is also in a negative relationship, and the air quality condition is improving. In terms of industrial structure, the regression results of the proportion of the output value of the secondary industry to GDP (SEC) and the value added of the secondary industry (SAV) are not significant; in terms of meteorological factors, the influence of each factor on urban air quality is basically insignificant, but the overall relationship is negative, the urban air pollutant concentration is decreasing, and the air quality condition is improving. The coefficient of the effect of precipitation on it is basically 0, and only O3 is significantly negative at 1% level.

The regression results of Central Guizhou City Cluster show that the coefficients of dummy variables of urban agglomeration construction, PM2.5, PM10 and SO2, are significantly negative at the 5% level, and AQI, NO2, CO and O3 are significantly negative at the 10% level, indicating that urban agglomeration construction significantly reduces the concentration of urban air pollutants, and the coefficients of dummy variables of urban agglomeration construction, AQI, are negatively related, and the urban air quality condition is to The coefficient AQI of urban cluster construction dummy variable is negatively related, and the urban air quality condition is improving.

From the regression results of the control variables, in terms of socio-economic factors and meteorological factors, the effects of each factor on urban air quality are not significant. Among the control variables, the share of secondary industry output in GDP (SEC) and annual average temperature (TEM) have a positive relationship with the explanatory variables, while the other control variables and explanatory variables have a negative relationship with each other.

The regression results of Central Chengdu-Chongqing City Cluster show that the coefficients of dummy variables of urban agglomeration construction AOI, PM10, PM2.5, SO2, and CO are significantly negative at the 1% level, indicating that urban agglomeration construction significantly reduces the concentrations of urban PM10, PM2.5, SO2, and CO, and the air quality index (AQI) is significantly better; while the regression results of NO2 and O3 are not significant.

From the regression results of the control variables, the socio-economic factors, the socio-economic development level has a significant effect on the urban AQI, PM10, PM2.5 and CO concentration with negative coefficients, which indicates that the socio-economic development level significantly reduces the urban PM10, PM and CO concentration, and the air quality index (AQI) has a significant trend to become better. Among the industrial structures, the effects of the proportion of the output value of the secondary industry to GDP on the concentrations of AOI, PM10, PM2.5, SO2, NO2, and CO were all significant and positive at the 1% level, indicating that the proportion of the output value of the secondary industry to GDP significantly increased the concentrations of PM10, PM2.5, SO2, NO2, and CO, and the AQI showed a significant worsening trend. In terms of meteorological factors, the effect of annual average temperature on AOI, PM10, PM2.5 is significantly positive, indicating that annual average temperature significantly increases the concentration of PM10, PM2.5, and AQI is significantly worse; the effect of annual average wind speed on AOI, PM10, PM2.5, SO2 is significantly negative, indicating that annual average temperature significantly decreases the concentration of PM10, PM2.5, SO2, and AQI. SO2concentrations, and the air quality index (AQI) showed a significantly better trend.

From the above regression results also untested: in general, the construction of urban agglomerations generally reduces the concentrations of urban air pollutants, urban air quality conditions are on a positive trend .From the results of the inclusion of control variables, the socio-economic factors of the Central Yunnan and Central Chengdu-Chongqing urban agglomerations basically show a trend towards lower urban air pollutant concentrations, Urban air quality conditions are improving. while the effect of socio-economic factors on urban air pollutant concentrations in the Central Guizhou urban agglomeration is insignificant. On the other hand, the effect of meteorological factors on urban air pollutant concentrations in the Central Yunnan and Central Guizhou urban agglomerations is insignificant, while the effect of meteorological factors in the Central Chengdu-Chongqing urban agglomeration on urban air pollutant concentrations is largely positive. This indicates that meteorological factors significantly augment the concentrations of urban air pollutants. Since the Yunnan-Central and Guizhou-Central urban agglomerations are located in the Yunnan-Guizhou plateau region, due to the influence of topographical conditions, air pollutants are easily dispersed and meteorological factors have little influence on them, while the Central Chengdu-Chongqing urban agglomeration is located in the Sichuan basin, where air pollutants cannot be easily dispersed and this agglomeration is more influenced by meteorological factors. Note that topographical conditions have a non-negligible impact on urban air quality.

### Test analysis

#### Parallel trend test

An important prerequisite for the validity of the difference-in-difference method is to satisfy the parallel trend assumption, i.e., assuming there is no urban cluster construction policy, the trends of air quality in urban cluster cities and other non-urban cluster cities should be consistent. In this paper, we introduce a cross term of policy control variables and time control variables for parallel trend test, in which no other variables are added to ensure that the experimental results are only influenced by the one policy of urban cluster construction. In order to test this, this paper uses regression method for parallel trend test, selecting air quality index (AQI) and setting four time dummy variables: a value of 1 when the policy is implemented 2 years before, 0 otherwise; a value of 1 when the policy is implemented 1 year before, 0 otherwise; a value of 1 when the policy is implemented this year, 0 otherwise. The following model was constructed:

3 $$Y_{it} = \alpha_{0} + \beta_{1} before2_{t} + \beta_{2} before1_{t} + \beta_{3} currecnt_{t} + \beta_{4} after1_{t} + \alpha_{1} X_{it} + \mu_{i} + \lambda_{t} + \varepsilon_{it}$$

The results in Table 6 show that the coefficients of the Central Yunnan Urban Agglomeration policy were not significant in the first 2 years of implementation and significant in the year before and 1 year after implementation, indicating that the Central Yunnan City Cluster had an impact on air quality before the formal approval. The coefficients of the Central Guizhou City Cluster policy were not significant in the first 2 years and the first 1 year of implementation, and systematic differences occurred over time after the policy implementation. The coefficients before and after the implementation of the Central Chengdu-Chongqing City Cluster policy are both significantly positive at the 1% level, and the coefficient in the year of policy implementation is significantly negative at the 5% level, indicating that the Central Chengdu-Chongqing City Cluster policy has an impact on air quality. In order to enter the city cluster planning area, each local government pays more attention to urban air quality and ecological environment in the preparation and approval stages of city cluster construction, and the air pollution control is stronger, and the air quality has been effectively improved, and the city cluster policy has a certain degree of foresight. In addition, the implementation of the policy has a certain lag, and the policy may not necessarily produce effects soon after implementation. The assumption of parallel trend is valid. Therefore, this paper considers that the preconditions for using DID are satisfied.

**Table 6:** Impact of urban agglomeration construction on air quality: parallel trend test.

	Central Yunnan City cluster	Central Guizhou City cluster	Central Chengdu-Chongqing City cluster
	AQI	AQI	AQI
before2	0.492	0.518	12.350***
	(0.37)	(0.36)	(10.41)
before1	− 4.977***	2.284	13.290***
	(− 3.70)	(1.57)	(11.20)
current	0.268	− 3.185**	11.525***
	(0.20)	(− 2.19)	(9.72)
after	− 4.857***	2.060	− 2.930**
	(− 3.61)	(1.42)	(− 2.47)
Constant	49.367***	47.576***	64.002***
	(63.59)	(56.66)	(93.46)
Sample sizes	96	96	132
R-squared	0.276	0.153	0.735
Number of id	16	16	22
Controls	No	No	No
Year effect	Yes	Yes	Yes

*, **, and *** represent 10%, 5%, and 1% significance levels, respectively; t-values in parentheses.

#### Robustness test PSM–DID

The endogeneity problem due to variable selection bias has been avoided by the DID in the previous section. Next, the propensity score matching method (PSM) was used to eliminate the sample selection bias by selecting other non-urban cities with similar development characteristics to the urban cluster cities as the control group.

The test results in Table 7 show that after using the PSM–DID method, the urban cluster construction still significantly reduces the urban air quality pollutant concentration and the urban air quality condition also shows a significantly better trend. This further supports the empirical findings of this paper. The standard errors estimated by the PSM–DID are all reduced compared to the standard errors of the ordinary DID, and the regression coefficients of the interaction term DID are much larger than those of the ordinary DID estimates. Overall, the PSM–DID estimates are better and more reliable, and the robustness test is passed. Urban cluster construction has an impact on urban air quality, and for the three major urban clusters selected in this paper, urban cluster construction significantly reduces urban air quality pollutant concentrations and urban air quality index.

**Table 7:** Influence of urban agglomeration construction on air quality: PSM–DID test.

	(1)	(2)	(3)	(4)	(5)	(6)	(7)
	AQI	PM10	PM2.5	SO2	NO2	CO	O3
DID	− 8.035***	− 16.406***	− 9.380***	− 6.540***	0.259	− 0.136***	2.531
	(− 3.81)	(− 5.45)	(− 4.77)	(− 6.13)	(0.29)	(− 4.10)	(1.26)
Constant	75.186***	74.001***	45.761***	17.234***	28.869***	0.901***	79.280***
	(46.87)	(32.32)	(30.62)	(21.24)	(41.96)	(35.89)	(51.95)
Sample sizes	132	132	132	132	132	132	132
R-squared	0.118	0.214	0.173	0.256	0.001	0.134	0.014
Number of id	22	22	22	22	22	22	22
Controls	No	No	No	No	No	No	No
Year effect	Yes	Yes	Yes	Yes	Yes	Yes	Yes

*, **, and *** represent 10%, 5%, and 1% significance levels respectively; t-values in parentheses.

#### Heterogeneity test

To examine the heterogeneity of the impact of city cluster construction on urban air quality, this paper further performs double differencing for urban agglomerations separately and non-urban agglomerations combined. The results are shown in Table 8.

**Table 8:** Influence of urban agglomeration construction on air quality: PSM–DID test.

	(1)	(2)	(3)	(4)	(5)	(6)	(7)
	AQI	PM2.5	PM10	SO2	NO2	CO	O3
DID	− 3.728*	− 4.208**	− 10.796***	− 3.710***	− 3.750***	− 0.125***	1.988
	(− 1.89)	(− 2.37)	(− 4.44)	(− 3.49)	(− 3.25)	(− 3.87)	(1.09)
PCG	0.000	0.000	0.000*	− 0.000	0.000***	0.000***	0.000
	(1.43)	(0.49)	(1.79)	(− 0.81)	(5.43)	(3.65)	(0.70)
SEC	0.862***	0.720***	1.039***	0.305***	0.321***	0.008***	0.223***
	(12.31)	(11.41)	(12.02)	(8.08)	(7.82)	(6.60)	(3.45)
SAV	0.002***	0.002***	0.002***	− 0.000	0.002***	0.000	− 0.000
	(4.63)	(3.91)	(3.18)	(− 0.77)	(7.15)	(0.09)	(− 0.01)
TEM	0.002	0.252	0.450	0.013	− 0.206	0.012***	0.399*
	(0.01)	(1.22)	(1.59)	(0.11)	(− 1.53)	(3.24)	(1.88)
PRE	0.000	0.001**	0.001**	0.001***	0.000	0.000	− 0.001***
	(0.29)	(2.38)	(2.44)	(2.98)	(0.25)	(0.86)	(− 4.08)
WIN	− 8.356***	− 6.195***	− 10.002***	1.576***	− 4.428***	− 0.010	0.470
	(− 8.61)	(− 7.09)	(− 8.35)	(3.01)	(− 7.79)	(− 0.64)	(0.52)
Constant	51.027***	11.632*	24.678***	− 11.426***	22.413***	0.156	75.658***
	(7.26)	(1.84)	(2.84)	(− 3.02)	(5.44)	(1.35)	(11.65)
Sample sizes	282	282	282	282	282	282	282
R-squared	0.547	0.495	0.548	0.290	0.561	0.324	0.141
Controls	No	No	No	No	No	No	No
Year effect	Yes	Yes	Yes	Yes	Yes	Yes	Yes

*, **, and *** represent 10%, 5%, and 1% significance levels respectively; t-values in parentheses.

The results showed that the construction of urban agglomerations significantly reduced the concentrations of urban air pollutants except for O3, and in addition the air quality index also showed a significant inverse relationship, the construction of urban agglomerations significantly reduced the urban air quality index, and the air quality condition showed a better trend.

In addition, socio-economic factors, the level of socio-economic development (PCG) has a significant effect on the concentrations of PM10, NO2, and CO in the city. Among the industrial structures, the effect of secondary industry output value as a percentage of GDP (SEC) on AOI and urban air quality pollutants was significantly positive at the 1% level, indicating that secondary industry output value as a percentage of GDP (SEC) significantly increased the concentration of urban air quality pollutants and air quality index (AQI), and the effect of secondary industry value added (SAV) on the concentration of AOI, PM10, PM2.5, NO2, CO The effects of secondary industry value added (SAV) on AOI, PM10, PM2.5, NO2, were all significantly positive at the 1% level, indicating that secondary industry value added (SAV) significantly increased the concentrations of PM10, PM2.5, NO2 and AQI; for meteorological factors, the effects of annual average temperature (TEM) on CO and O3 were significantly positive, indicating that annual average temperature significantly increased the concentrations of CO and O3, and the effects of annual precipitation (PRE) on The effects of annual precipitation (PRE) on PM10, PM2.5, and SO2 were all significantly positive at the 1% level, indicating that annual precipitation significantly increased the concentrations of PM10, PM2.5, and SO2, and the effects on O3 were significantly negative at the 1% level, indicating that O3 significantly decreased the concentration of O3, and the annual average wind speed significantly decreased the concentrations of PM2.5, PM10, SO2, NO2, and AQI.

### Analysis of air quality pollutant concentration distribution patterns

**Figure 2:**
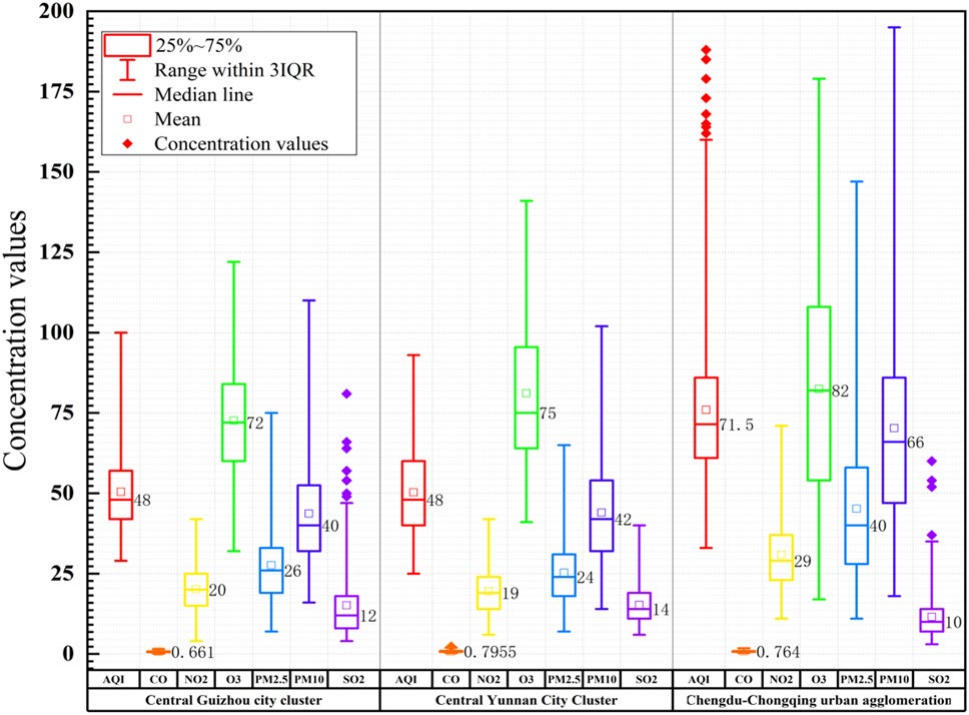
BOX distribution diagram of air quality pollutants in various urban agglomerations from 2015 to 2020. The charts were plotted using Origin 2022.

**Figure 3:**
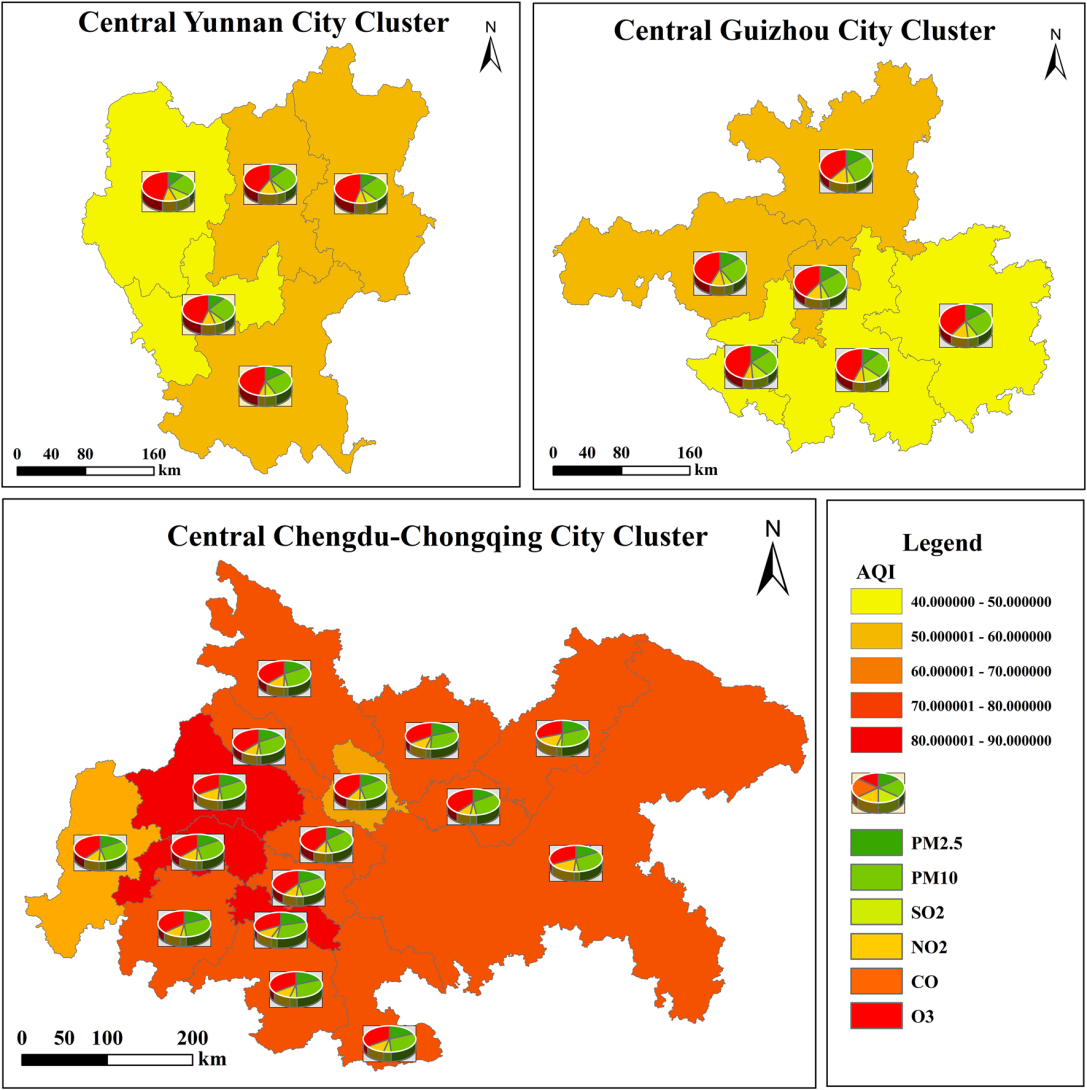
Spatial distribution of average air quality pollutants by urban agglomerations in 2015–2020.The maps in this study were processed and plotted by ArcGIS 10.8. The map images we use come from Environmental Sci-ences, Chinese Academy of Sciences (http://www.resdc.cn/).

Using the average values of air quality pollutant concentrations in cities within each city cluster from 2015 to 2020, the BOX distribution of air quality pollutant concentrations in each city cluster is plotted (Fig. 2) and the spatial distribution of average air quality pollutant values in each city cluster from 2015 to 2020 (Fig. 3) and it was found that the distribution pattern is unstable, and there were large differences in air quality pollutant concentrations between different city groups.

The urban air quality distribution pattern shows two patterns according to the extent of air quality pollutant concentration dispersion and the distribution pattern of the urban agglomerations. As can be seen from Fig. 2, in this context, the Central Chengdu-Chongqing urban agglomeration is a high-value upward bias type, with high concentrations of air quality pollutants; the Central Yunnan and Central Guizhou urban agglomerations are a medium value upward bias type, with medium air quality indices in this type of urban agglomerations. The common point between these two types is that the number of cities above the median value is inferior to the number below the median value, indicating an obvious air pollution dispersion effect within the urban agglomerations, and showing that few heavily polluted cities have a greater impact on the air quality of the urban agglomerations, and key air pollution remediation has not achieved significant results. Overall, the median position of the annual assessment value distribution is clearly skewed towards the upper quartile, and the number of cities above the median value is superior to the number below it. As can be seen from Fig. 3, the spatial differences in air quality among urban agglomerations in the southwest are obvious. Specifically, the air pollution in the Chengdu-Chongqing urban agglomeration is the most serious, and the concentration values of O3 are the highest in all three urban agglomerations.

## Conclusion and policy implications

### Conclusion

In recent years, urban agglomerations have been planned and constructed, and their number has gradually increased and their coverage has been expanded. This paper verifies the issue of the impact of the construction of three major urban agglomerations on air quality based on PSM–DID. The study finds that:

- Through the empirical analysis and testing, it is found that the construction of urban agglomerations significantly improves urban air quality in the whole sample. In addition, the effect of urban cluster construction on air quality was found to be forward-looking and lagging in the testing process.
- In terms of the impact factors, the socio-economic aspects of urban agglomeration construction, the level of economic development of a region has a certain influence on urban air quality. Industrial structure significantly deteriorates urban air quality in urban agglomerations. Meteorological factors significantly affect the air quality of urban cluster cities. Among them, the average annual rainfall of a city has an impact on urban air quality, as rainfall improves urban air quality by removing particulate matter from the atmosphere through wet deposition. The average annual temperature significantly increases the concentration of air pollutants in cities. Areas with good temperature conditions have frequent economic activities, large population gatherings, increased population density, and further expansion of demand, which will accelerate the further development of urban infrastructure construction, leading to increased air pollution and deterioration of urban air quality. The annual average wind speed can reduce the concentration of air pollutants, the wind will expand the range of air pollutants, but at the same time can also reduce the degree of pollution. The higher the wind speed, the lower the concentration of air quality pollutants and the better the air quality condition.
- Urban agglomeration construction has spatial heterogeneity on air quality impacts. In general, the construction of urban agglomerations generally reduces the concentration of urban air pollutants and improves urban air quality. The influence of meteorological factors on urban air pollutant concentrations is not significant in the Yunnan-Central and Qian-Central urban agglomerations, while the influence of meteorological factors on urban air pollutant concentrations in the Chengdu-Chongqing urban agglomeration is basically significant and positive, because the Yunnan-Central and Qian-Central urban agglomerations are located in the Yunnan-Guizhou plateau area, the air pollutants can be easily dispersed due to the topographic conditions, and meteorological factors have little influence on them, while the Chengdu-Chongqing urban agglomeration is located in the Sichuan basin. air pollutants are not easy to disperse and are more influenced by meteorological factors. The topographic conditions of urban air quality have a non-negligible influence on pollutant concentrations. In terms of socio-economic factors, the construction of Chengdu-Chongqing urban agglomeration has a greater impact on urban air quality, while the construction of Central Yunnan urban agglomeration and Central Qian urban agglomeration has a smaller impact on urban air quality, because the development of Chengdu-Chongqing urban agglomeration is relatively mature, while the other two urban agglomerations are still under "cultivation and development", resulting in different impacts on the development of each urban agglomeration.
- From the perspective of urban air quality pollutant concentration distribution patterns, urban agglomerations have unstable air quality pollutant concentration distribution patterns. And there are large differences in air quality pollutant concentrations in different urban agglomerations. There is an obvious air pollution diffusion effect within the urban agglomerations, and a few severely polluted cities have a greater impact on the air quality of urban agglomerations, and the key air pollution remediation has not achieved significant results. Urban clusters are beginning to show air quality pollution integration, and inter-regional cooperation should be strengthened to promote air quality pollution management policies as a whole.

### Policy implications

These findings suggest some policy implications for the development of urban agglomerations

- Improve the standard of urban cluster planning approval. In order to enter the scope of urban agglomeration planning, each local government pays more attention to urban air quality and ecological environment in the preparation and approval stage of urban agglomeration construction, and the air pollution control is stronger, and the air quality has been effectively improved.
- Implementation of high-quality development strategy. Areas with high level of economic development invest more in urban air quality management, and urban air quality has been better improved. The construction of urban clusters in pursuit of rapid economic growth will inevitably lead to large amounts of energy consumption and air pollution, and the "living effect" of urbanization caused by population clustering, resulting in deterioration of air quality. The concept of high-quality development should be implemented while developing the economy.
- Policy makers should also make efforts to optimize the industrial structure of urban clusters. The secondary industry is characterized by high energy consumption and high emissions. Urban agglomerations with high level of secondary industry development will increase the direct emission of pollutants into the atmosphere, which will increase air pollution in urban agglomerations and worsen urban air quality. Therefore, the overall layout of the regional industrial structure, to promote industrial clustering, through centralized pollution control and thus reduce the cost of pollution control, to achieve the scale benefits of centralized pollution control.
- The development plan of urban clusters should be made according to local conditions. According to the principle of optimal balance of production, living and ecological space, the scale of cities and urban agglomerations should be strengthened while their spatial structure is reasonably planned with production and living functions.

## Data Availability

The datasets generated and analyzed in this study are available from the corresponding author on reasonable request.
